# Editorial: Understanding the RNA Species in the Extracellular Vesicles of Multiple Myeloma

**DOI:** 10.3389/fonc.2022.946160

**Published:** 2022-06-23

**Authors:** Maoshan Chen, Rong Xu, Jing Zhang, Andrew Spencer, Richard Simpson

**Affiliations:** ^1^ Laboratory of Radiation Biology, Department of Blood Transfusion, Laboratory Medicine Centre, The Second Affiliated Hospital, Army Medical University, Chongqing, China; ^2^ Nanobiotechnology Laboratory, Australian Centre for Blood Diseases, Monash University/Alfred Health, Melbourne, VIC, Australia; ^3^ Department of Oncology, Tongji Hospital, Tongji Medical College, Huazhong University of Science and Technology, Wuhan, China; ^4^ Malignant Haematology and Stem Cell Transplantation Service, Alfred Health-Monash University, Melbourne, VIC, Australia; ^5^ Department of Biochemistry and Chemistry, La Trobe Institute for Molecular Science (LIMS), School of Agriculture, Biomedicine and Environment, La Trobe University, Melbourne, VIC, Australia

**Keywords:** Multiple myeloma, Extracellular vesicles, Exosomes, Liquid biopsy, Tumour microenvironment

Multiple myeloma (MM) is a multi-focal plasma cell malignancy that develops at diverse sites throughout the bone marrow (BM) compartment (1). Despite advances in therapy in the past 15 years, MM remains incurable with a remitting relapsing clinical course and the invariable development of disease that is refractory to all available therapies. Emerging evidence has implicated extracellular vesicles (EVs), heterogeneous nano-sized lipid-bound organelles shed by most cells into the extracellular space (2), in MM pathogenesis, including angiogenesis, osteolysis, immune suppression environment and drug resistance (3). Based on their size, EVs comprise three major classes – exosomes (30 ~150 nm, of endocytic origin), shed midbody remnants (which arise from symmetrical abscission of the midbody during the late stage of cytokinesis) and shed microvesicles (50 ~ 1500 nm, aka microparticles, which arise from blebbing of the plasma membrane), and can transfer DNA fragments, RNAs, proteins, lipids and metabolites to both neighbouring and distant cells (2).

This Research Topic aims to bring together clinical and basic scientists, and summarise our understanding of the roles of EV RNA molecules (e.g., microRNA, mRNA, circular RNA, long non-coding RNA) in MM. And although this area has been studied in many solid tumours, it is a relatively new area of investigation in MM. True to its aim, this special issue has included two review articles that highlight some of the most recent advances regarding the identification, characterization, and function of EV RNAs in MM pathogenesis and potential translational applications.

The BM tumour microenvironment (TME) is a critical mediator of growth and survival for MM plasma cells. In this context, Khalife et al. for the first time have demonstrated the effects of MM-EV-derived RNAs on a range of TME compartments (e.g., hematopoietic cells, mesenchymal stromal cells (MSC), endothelial cells, immune cells, osteoclasts, and osteoblasts) all of which are understood to play a role in MM pathology. These include plasma cell proliferation, escape from immune surveillance, angiogenesis and lytic bone disease (Khalife et al.). The tumour suppressor miR-15a can be transferred by the MM-BM-MSC EVs into the MM plasma cells and activates the AKT pathway, promoting proliferation, survival, and migration of MM cells (4, 5). Similarly, miR-10a and the long non-coding RNA *LINCOO461* can be transferred to MM cells *via* MSC EVs and induce the proliferation of MM cells (6, 7). EV RNAs also play an important role in the inhibition of osteoblast development, osteoclast differentiation, and their bone resorption activity, such as miR-103-3p (8), miR-129 (9) and DKK-1 mRNA (10).

In another contribution, Reale et al. have summarised the translational applications of MM-derived EV RNAs, including in relation to drug resistance, liquid biomarkers, and therapeutic strategies (Reale et al). By comparing the RNA profiles of EVs isolated from the serum and plasma of MM patients and healthy individuals, several RNAs have been suggested as potential clinical biomarkers, including microRNAs (e.g., miR-155, miR-34a, let-7, miR-20a, miR-103a, miR-185, miR-425, miR-4505, miR-4741), mRNA transcripts (e.g., PSMA3), and long non-coding RNAs (e.g., PRINS, PSMA3-AS1). Regarding potential therapeutic applications, c-Myc and TGF-β1 were targeted with RNA-armed EVs or engineered EV-like nanoparticles for the transfer of siRNAs, shRNAs, and antisense oligonucleotides into the lymphoma models (11–13). However, proof of concept of EV-RNA-engineered therapy for MM is still warranted. In this Research Topic, both Khalife et al. and Reale et al. agreed that EVs can be purified from both biofluids of MM patients and cell culture supernatants; however, the standardization of isolation protocols and criteria for characterizing purified MM-derived EVs is still essential for lab-to-lab comparisons (Khalife et al.; Reale et al.).

In conclusion, the articles collected in this Research Topic provide a series of insightful datasets to better understand the biological functions and translational applications of RNA molecules in the MM-derived EVs. However, further studies investigating the roles of MM-EV RNA *in vivo* are required. We trust that the high-quality information published in this issue of Frontiers in Oncology will be of interest to researchers in the fields of MM pathogenesis, biomarkers, and therapeutics.

## Author Contributions

MC –prepared the first version of the editorial. MC, RX, JZ, AS and RJ - discuss the editorial content and revised the final editorial text. All authors contributed to the article and approved the submitted version.

## Conflict of Interest

The authors declare that the research was conducted in the absence of any commercial or financial relationships that could be construed as a potential conflict of interest.

## Publisher’s Note

All claims expressed in this article are solely those of the authors and do not necessarily represent those of their affiliated organizations, or those of the publisher, the editors and the reviewers. Any product that may be evaluated in this article, or claim that may be made by its manufacturer, is not guaranteed or endorsed by the publisher.
